# Diagnostic delay in rare diseases: data from the Spanish rare diseases patient registry

**DOI:** 10.1186/s13023-022-02530-3

**Published:** 2022-11-17

**Authors:** Juan Benito-Lozano, Blanca López-Villalba, Greta Arias-Merino, Manuel Posada de la Paz, Verónica Alonso-Ferreira

**Affiliations:** 1 Institute of Rare Diseases Research (IIER), Instituto de Salud Carlos III, 28029 Madrid, Spain; 2 Universidad Nacional de Educación a Distancia (UNED), 28015 Madrid, Spain; 3 Preventive Medicine and Public Health, Hospital Clínico Universitario de Valladolid (HCUV), 47003 Valladolid, Spain; 4 Centre for Biomedical Network Research On Rare Diseases (CIBERER), Instituto de Salud Carlos III, 28029 Madrid, Spain

**Keywords:** Rare diseases, Diagnostic delay, Time to diagnosis, Patient registry, Spain, Public health

## Abstract

**Background:**

According to the International Rare Diseases Research Consortium (IRDiRC), a known rare disease (RD) should be diagnosable within a year. This study sought: firstly, to ascertain how long it takes to obtain the diagnosis of a RD in Spain, along with its associated time trend; and secondly, to identify and measure diagnostic delay (defined by the IRDiRC as any period exceeding a year) by reference to the characteristics of RDs and the persons affected by them.

**Methods:**

Using data sourced from the Spanish Rare Diseases Patient Registry, we performed a descriptive analysis of the time elapsed between symptom onset and diagnosis of each RD, by sex, age and date of symptom onset, and type of RD. We analysed the time trend across the period 1960–2021 and possible change points, using a Joinpoint regression model and assuming a Poisson distribution. The multivariate analysis was completed with backward stepwise logistic regression.

**Results:**

Detailed information was obtained on 3304 persons with RDs: 56.4% had experienced delay in diagnosis of their RDs, with the mean time taken being 6.18 years (median = 2; IQR 0.2–7.5). Both the percentage of patients with diagnostic delay and the average time to diagnosis underwent a significant reduction across the study period (p < 0.001). There was a higher percentage of diagnostic delays: in women (OR 1.25; 95% CI 1.07–1.45); in cases with symptom onset at age 30–44 years (OR 1.48; 95% CI 1.19–1.84): and when analysed by type of RD, in mental and behavioural disorders (OR 4.21; 95% CI 2.26–7.85), followed by RDs of the nervous system (OR 1.39; 95% CI 1.02–1.88).

**Conclusions:**

This is the first study to quantify time to diagnosis of RDs in Spain, based on data from a national registry open to any RD. Since over half of all persons affected by RDs experience delay in diagnosis, new studies are needed to ascertain the factors associated with this delay and the implications this has on the lives of patients and their families.

**Supplementary Information:**

The online version contains supplementary material available at 10.1186/s13023-022-02530-3.

## Background

Rare diseases (RDs) as a whole affect 3.5–8% of the population and thus represents a public health problem [1, 2]. Since these are very diverse diseases in terms of presentation and cause, obtaining the diagnosis of a RD in the shortest time possible poses a great challenge to health professionals and society. Despite there being increasingly more knowledge on the subject, more resources allocated and new technologies developed, diagnosis of most of these diseases continues to be complex. Hence, there is often a delay in diagnosis, which entails suffering for patients and their families alike, a high cost for the health system, as well as a possible exacerbation of the undiagnosed disease. Some of the causes of this delay in obtaining a diagnosis are linked to the lack of scientific knowledge on each of the thousands of RDs, the presence of nonspecific symptoms, the point in time when the patient seeks medical advice, the availability of diagnostic tests, and the difficulties facing specialists when it comes to making a complete diagnosis without waiting or losing time [3, 4].

Reducing diagnostic delay is a priority in research and socio-healthcare policies, as well as an ethical obligation. Among its goals for 2027, the International Rare Diseases Research Consortium (*IRDiRC*) requires that all known RDs be diagnosed within a maximum of one year from the date on which medical advice on the symptoms is first sought [5]. Hence, any case where a disease is diagnosed after more than a year will be deemed to qualify as a diagnostic delay.

Despite its importance, there is scant scientific evidence on delay in diagnosis of RDs [6], and what information is available on this topic tends to be furnished by patient associations. At a European level, EURORDIS indicates that “25% of patients had to wait between 5 and 30 years from early symptoms to confirmatory diagnosis of their disease. Also, 40% of patients first received an erroneous diagnosis, others received none” [7]. The Spanish Rare Disease Federation (*Federación Española de Enfermedades Raras*, *FEDER*) quantified the mean time taken by patients to obtain a diagnosis as 4 years, indicating that 20% took 10 years or more [8].

Both in the scientific world and in patient associations, the expression “diagnostic odyssey” [9, 10] is used to refer, not only to the delay in diagnosis, but also to this journey in which there may have been interminable referrals to specialists, interruptions in the search, or even erroneous diagnoses, at times amounting to a whole diagnostic process. Delay in diagnosis of RDs thus involves consequences, not only for patients but also for their families, such as receiving no support or treatment, or inappropriate treatment. This may in turn have repercussions, such as exacerbation of the disease, or effects on psychological [11], social [12], occupational [13], financial [14], educational [15], or family aspects [16], among others.

The aim of this study was thus: firstly, to ascertain the time taken to obtain the diagnosis of a RD in Spain, along with its associated time trend; and secondly, to characterise diagnostic delay on the basis of the IRDiRC goal, by reference to the main characteristics of RDs and the persons affected by them.

## Methods

The data were drawn from the Rare Diseases Patient Registry at the Institute of Health Carlos III (*Instituto de Salud Carlos III, ISCIII*) [17]. The aim of the Registry was to provide researchers, health professionals and groups of patients with a greater level of knowledge on RDs, in order to foster research, increase these diseases’ visibility, and favour decision-making for appropriate healthcare planning and correct distribution of resources [18]. There are two ways in which entries can be made on this register: on the one hand, voluntarily, by the patients themselves or their families; and, on the other, by any professional participants in research networks or medical societies, with wide-ranging experience in a specific RD group, which have an agreement with the ISCIII to develop this record within the Rare Diseases Patient Registry. Patients registered are required to provide informed consent and clinical and/or genetic reports on their diagnoses, which are reviewed by the staff of the Institute of Rare Diseases Research (Instituto de Investigación de Enfermedades Raras, IIER), who then decide which RD is involved and code it accordingly. RDs are coded in the Registry in accordance with the respective classification systems, namely, ORPHAcode, International Classification of Diseases-Tenth Revision (ICD10) and, where possible, Online Mendelian Inheritance in Man (OMIM). If the information supplied does not suffice to specify the RD from which the patient suffers, the process is halted and new clinical reports are sought from the patient and/or his/her physician. When a suspected case of RD exists, the procedure of entry is the same than in confirmed ones: any patient can register voluntarily or any clinician can register a patient. In both cases, all the clinical information available should be provided to be studied. In addition, the Registry includes patient-reported data and questionnaires with different purposes, one of which is specifically targeted at the process of searching for the diagnosis of the RD. In this study, we included all patients entered on the Registry at 1 January 2022, who: had a confirmed diagnosis of a RD, along with detailed information about their diagnostic process; were of any age, sex or vital status (alive or deceased); and were Spanish residents at the moment of the enrolment in the registry.

The main variable of analysis -time to diagnosis- was calculated as the time elapsed (in years) from the date of symptom onset until the date when diagnosis of a RD was obtained. Based on the goal set by the IRDiRC, delay was defined as more than one year in this diagnosis process. In cases where the date of symptom onset or diagnosis was not available but the patient’s age at these points in time was in fact known, time to diagnosis was calculated on the basis of this information. In the case of prenatal diagnoses and pre-symptomatic diagnoses, diagnosis was assumed to have made at birth, so that the date of symptom onset and diagnosis coincided, and time to diagnosis was therefore 0 years.

We performed a descriptive analysis of the following variables of; sex;, patient’s age at symptom onset (under 15, 15–29, 30–44, and 45 years and over); decade of symptom onset (1960 to 2021); Autonomous Region “*Comunidad Autónoma*” (the 17 main regions on the basis of which public health services are organised in Spain); and type of RD (specific RDs, RD groups, or large groups in terms of the organ or principal system affected as per ICD10 criteria). See Additional file 1 to check the complete list of included RD and their ORPHAcodes.

For the time-trend analysis of the percentage of patients affected by diagnostic delay and the average time needed to achieve at a diagnosis, Annual Percent Changes (APC) were calculated using a Joinpoint regression model and assuming a Poisson distribution. The multivariate analysis was completed with a backward stepwise logistic regression, with likelihood ratio, in order to ascertain the odds ratio (OR) or risk of diagnostic delay according to the above-mentioned variables, taking the following as reference groups: men (sex); < 15 years of age (symptom onset); period 2010–2021 (decade of diagnosis); and endocrine, nutritional and metabolic diseases (type of RD). All analyses were performed using the SPSS v22 and Joinpoint v4.9.0.0 statistics programmes.

## Results

**Fig. 1:**
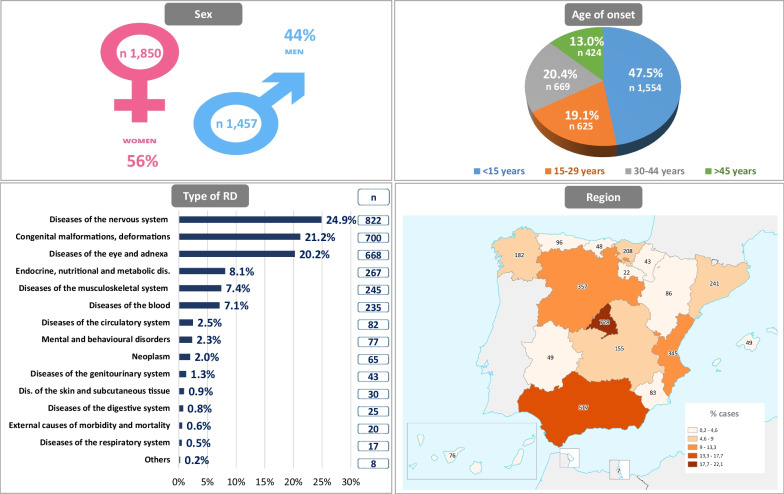
Sample characteristics

Information on time to diagnosis of RDs was obtained for 3,349 patients. Those cases with data that were incomplete or not in line with the natural history of the disease, were excluded. As a consequence of this screening process, the results reported below correspond to a total of 3,304 patients. Sample characteristics of patients included in this study are shown in Fig. 1.

**Fig. 2:**
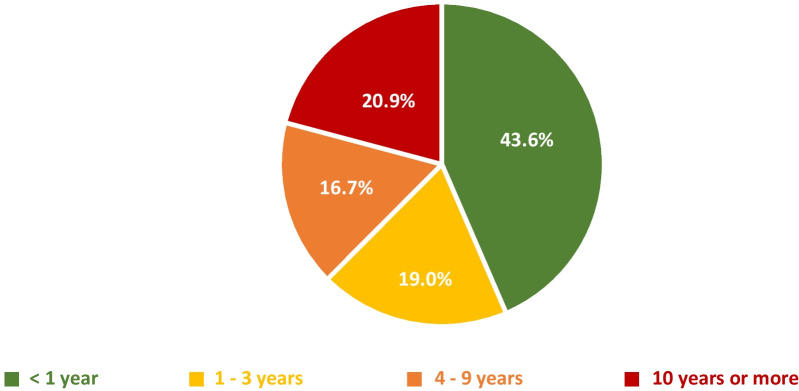
Time to diagnosis of RDs in Spain

Mean time to diagnosis of a RD in Spain was 6.18 years (median = 2; IQR 0.2–7.5). More than half of all persons affected by RDs experienced some delay in the diagnosis of their disease (Fig. 2): 56.4% of patients took over a year, 19.0% took 1 to 3 years, 16.7% took 4 to 9 years, and 20.9% waited for more than 10 years to be diagnosed.

Table 1 shows the percentage of persons who experienced a delay in diagnosis of their RD, by reference to examples of specific RDs or groups of RDs. Percentage delays of over 70% were observed in patients affected by diseases classified as mental and behavioural disorders, Usher syndrome, Sjögren’s syndrome, Behçet’s disease and cone-rod dystrophy. Special mention should be made of hereditary spastic paraplegias and post-polio syndrome which showed diagnostic delays in over 80% of patients.

**Table 1:** Percentage of people who have suffered diagnostic delays in Spain, according to type of RD (classified according to the organ or system mainly affected), along with examples of specific RDs or RD groups represented by more than 30 cases

Type of RD	RD or RD group	ICD10	ORPHA code	n		Diagnostic delay (%)	
				Total	Men–Women	Total (%)	Men–Women (%)
Diseases of the nervous system	Total	G00–G99		822	375–447	64.2	63.2–65.1
	Tarlov cyst	G58.9	65, 250	128	14–114	57.8	64.3–57.0
	Muscular dystrophies	G71.0	98, 473*	90	52–38	67.8	59.6–78.9
	Hereditary ataxias	G11	183, 518*	74	40–34	63.5	67.5–58.8
	Post-poliomyelitis syndrome	G14.0	2942	56	30–26	83.9	80.0–88.5
	Hereditary spastic paraplegia	G11.4	685*	53	33–20	88.7	84.8–95.0
	Amyotrophic lateral sclerosis	G12.2	803	36	22–14	52.8	59.1–42.9
	Myasthenia gravis	G70.0	589	35	15–20	34.3	20.0–45.0
Congenital malformations, deformations and chromosomal abnormalities	Total	Q00–Q99		700	308–392	48.4	45.8–50.5
	Arnold-Chiari malformation	Q07.0	1136, 268, 882	60	19–41	65.0	52.6–70.7
	Epidermolysis bullosa	Q81	304*	38	20–18	44.7	40.0–50.0
Diseases of the eye and adnexa	Total	H00–H59		668	349–319	62.9	57.6–68.7
	Retinitis pigmentosa	H35.5	791	384	185–199	65.1	61.1–68.8
	Stargardt disease	H35.5	827	72	35–37	56.9	48.6–64.9
	Usher syndrome	H35.5	886	55	32–23	76.4	71.9–82.6
	Cone-rod dystrophy	H35.5	1872	30	16–14	70.0	68.8–71.4
Endocrine, nutritional and metabolic diseases	Total	E00–E90		267	131–136	55.4	48.9–61.8
Diseases of the musculoskeletal system and connective tissue	Total	M00–M99		245	76–169	53.1	42.1–58.0
	Systemic sclerosis	M34	801*	48	10–38	56.3	70.0–52.6
	Behçet’s disease	M35.2	117	31	9–22	71.0	55.6–77.3
	Sjögren-Larsson syndrome	M35.0	816	30	3–27	73.3	33.3–77.8
Diseases of the blood and blood-forming organs and certain disorders involving the immune mechanism	Total	D50–D89		235	96–139	44.7	41.7–46.8
	Hereditary angioedema	D84.1	91, 378*	108	40–68	55.6	47.5–60.3
	Sarcoidosis	D86	797	50	25–25	26.0	28.0–24.0
Diseases of the circulatory system	Total	I00–I99		82	10–72	51.2	30.0–54.2
	Lymphangioleiomyomatosis	I89.8	538	46	**− 46	56.5	**− 56.5
Mental and behavioural disorders	Total	F00–F99		77	38–39	78.0	76.0–79.0
Neoplasms	Total	C00–D48		65	27–38	35.0	37.0–34.0
Diseases of the genitourinary system	Total	N00–N99		43	5–38	67.0	60.0–68.0
Diseases of the skin and subcutaneous tissue	Total	L00–L99		30	9–21	53.0	67.0–48.0
Total				3304	1454-1850	56.4	53.4-58.8

*Group of disorders

**RDs that mainly affect women

In contrast, lower percentages of diagnostic delay were recorded by patients affected by congenital malformations, deformities and chromosomal anomalies, due to diseases of the blood and haematopoietic organs or due to epidermolysis bullosa (less than 50% of patients experienced a delay). Of note here were rare cancers, myasthenia gravis and sarcoidosis, with delays recorded in less than 35% of cases (Table 1).

**Fig. 3:**
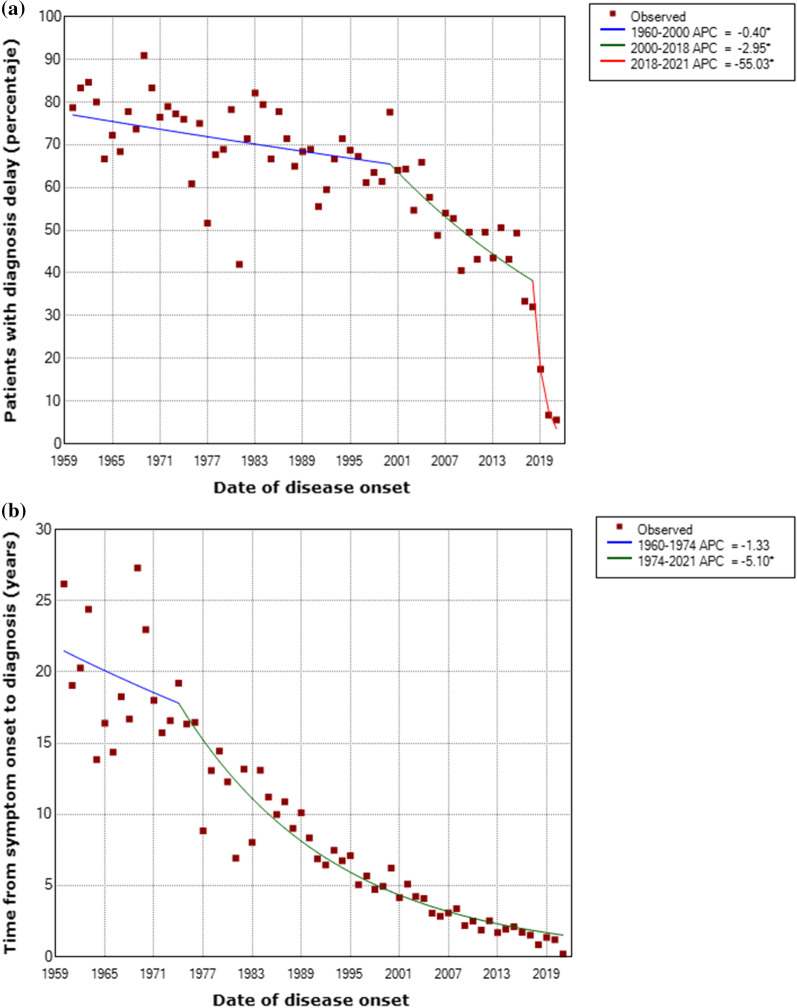
Time trend in diagnostic delay (**a**) and time to diagnosis **b** of RDs in Spain 1960–2021. *Indicates that the Annual Percent Change (APC) is significantly different from zero at the alpha = 0.05 level. Note: Patients with symptom onset before 1960 were included in that year

In terms of the time trend, the percentage of RD-affected patients who experienced diagnostic delays fell annually by 0.40% (APC) from 1969 until 2000 (p = 0.027), by 2.95% until 2018 (p < 0.001), and by even more from 2018 until 2021 (55.03%, p = 0.014; Fig. 3a). Similarly, time to diagnosis fell at an annual rate of 5.1% from 1974 to 2021 (p < 0.001; Fig. 3b).

When it comes to characterising diagnostic delays, Table 2 shows the results broken down by sex, age of symptom onset, decade of symptom onset, and type of disease (by ICD10 rubric). Greater diagnostic delays were observed among women (OR 1.25; 95% CI 1.07–1.45) and in cases where the age of symptom onset ranged from 30 to 44 years (OR 1.48; 95% CI 1.19–1.84). When RDs were broken down by organ or main system affected, mental and behavioural disorders (OR 4.21; 95% CI 2.26–7.85) and diseases of the nervous system (OR 1.39; 95% CI 1.02–1.88) had a higher risk of experiencing diagnostic delay. Compared to this, there was a lower risk of delay in diagnosis of rare cancers (OR 0.48; 95% CI 0.26–0.87), diseases of the blood, haematopoietic organs and other disorders that affect the immune mechanism (OR 0.64; 95% CI 0.44–0.94), and congenital malformations, deformities and chromosomal anomalies (OR 0.70; 95% CI 0.51–0.94). There were also differences according to decade of symptom onset, in that patients with symptom onset before 1979 had a fivefold higher risk of experiencing diagnostic delay (OR 5.27; 95% CI 4.00–6.94) than did those with symptom onset in the current period. The figures confirmed the fact that the more recent the date of symptom onset, the lower the percentage of patients affected by diagnostic delay. Lastly, it should be mentioned that no significant differences were found by patients’ home area or Autonomous Region (data not shown).

**Table 2:** Time to diagnosis and diagnostic delays for RDs in Spain, based on characteristics of the disease and people affected

Variable	Category	Time to diagnosis		Diagnostic delay (> 1 year)		
		n	Median (IQR)	Percentage	OR (95% CI)	p value
Sex	Men*	1454	5.6 (0.1–6.3)	53.4		0.005
	Women	1850	6.7 (0.2–8.5)	58.8	1.25 (1.07–1.45)	0.005
Age of onset of symptoms	< 15 years*	1554	7.7 (0.1–10)	55.2		NS
	15–29 years	625	7.1 (0.2–11)	61.8	1.19 (0.96–1.48)	NS
	30–44 years	669	4.1 (0.4–5.4)	59.0	1.48 (1.19–1.84)	< 0.001
	> 45 years	424	2.6 (0.2–3.1)	50.5	1.24 (0.96–1.60)	NS
Decade of symptom onset	2010–2021*	969	0.9 (0.1–2.4)	40.4		< 0.001
	2000–2009	983	1.8 (0.2–5.7)	56.5	1.95 (1.62–2.35)	
	1990–1999	503	3.0 (0.3–11.0)	65.0	2.90 (2.28–3.67)	
	1980–1989	376	6.0 (0.4–19.0)	69.7	4.60 (3.46–6.13)	
	Until 1979	431	13.1 (1–32.7)	74.0	5.27 (4.00–6.94)	
Type of RD	Endocrine, nutritional and metabolic diseases *	267	6.1 (0.3–6.3)	55.4		< 0.001
	Diseases of the nervous system	822	6.3 (0.6–7.9)	64.2	1.39 (1.02–1.88)	0.035
	Congenital malformations, deformations and chromosomal abnormalities	700	5.8 (0–6.5)	48.4	0.70 (0.51–0.94)	0.016
	Diseases of the eye and adnexa	668	7.6 (0–10.0)	62.9	1.03 (0.76–1.40)	NS
	Diseases of the musculoskeletal system and connective tissue	245	4.6 (0.3–5.5)	53.1	0.87 (0.60–1.27)	NS
	Diseases of the blood and blood-forming organs and certain disorders involving the immune mechanism	235	6.5 (0–7.8)	44.7	0.64 (0.44–0.94)	0.021
	Diseases of the circulatory system	82	5.1 (0.1–4.9)	51.2	0.70 (0.41–1.2)	NS
	Mental and behavioural disorders	77	7.3 (1.2–10.9)	77.9	4.21 (2.26–7.85)	< 0.001
	Neoplasm	65	4.1 (0.1–2.8)	35.4	0.48 (0.26–0.87)	0.015
	Diseases of the genitourinary system	43	6.3 (1–6.2)	67.4	1.64 (0.80–3.34)	NS
	Diseases of the skin and subcutaneous tissue	30	5.2 (0.1–7.8)	53.3	0.80 (0.36–1.74)	NS
	Diseases of the digestive system	25	2.1 (0–3.6)	48.0	0.83 (0.35–1.97)	NS
	External causes of morbidity and mortality**	20	0.1 (0–0.1)	0.0	0.00	NS
	Diseases of the respiratory system	17	1.8 (0.2–2.5)	41.2	0.78 (0.28–2.17)	NS
	Others	8	5.6 (0.4–6.8)	62.5	1.78 (0.40–7.88)	NS

*Reference groups

**People affected by Toxic oil syndrome, who were diagnosed less than one year from the onset of the epidemic in 1981

*IQR* interquartile range; OR odds ratio; *NS* no significant difference (p > 0.05)

## Discussion

This is first study to quantify diagnostic delay for all RDs in Spain, based on information sourced from a national registry open to any RD, something that gives it an advantage over other studies based on surveys of patients with self-reported RDs. The reason for this is that all persons entered on the Spanish RD Patient Registry have a clinical report which makes it possible, on the one hand, to validate that they are indeed affected by a disease which fulfils the criteria for being considered a RD, and on the other, to verify their dates of symptom onset and diagnosis.

The mean time to diagnosis of a RD in Spain is 6.18 years, similar to that estimated by other international studies [19, 20]. More than half of all RD patients experience diagnostic delays in Spain (56.4%), with this being somewhat higher than the result obtained in the ENSERio study of FEDER [8], which reported a figure of 49.7% in a sample of 1576 patients. A more detailed comparison shows that both studies highlight the seriousness of this problem, with very similar percentages of diagnostic delay: 18.9% (ENSERio [8]) versus 19% (our study) of patients took 1 to 3 years, 18.1% versus 16.7% took 4 to 9 years, and 18.7% versus 20.9% waited more than 10 years to obtain a diagnosis. This thus confirms that for a high percentage of persons affected by RDs in Spain, the IRDiRC goal of obtaining diagnosis in the first year of examination has not be achieved.

In the international context, only EURORDIS’ EurordisCare 2 study offers general data for a group of 8 RDs, indicating that 25% of patients took more than 5 years to obtain their respective diagnoses [7]. Other studies report the median time to diagnosis for some RDs of the nervous system, i.e., Friedreich ataxia (36 months) [21], ataxia-telangiectasia (12 months) [22] and Duchenne muscular dystrophy (12 months) [23]. Furthermore, a time to diagnosis of less than a year has been reported for amyotrophic lateral sclerosis (11.5 months) [24] and inflammatory demyelinating polyradiculoneuropathy (5 months) [25]. In spite of the fact that there are diagnostic criteria for post-polio syndrome, 83.9% of these patients took over a year to be diagnosed (our study). Early diagnosis of this disease is complicated by the need for differential diagnosis, and even more so in the older population, by the overlapping of symptoms with other usual comorbidities. [26]

Similarly, time to diagnosis has also registered median values of over a year for some rare metabolic diseases: alpha-1 antitrypsin deficiency (22.3 years) [27], Pompe disease (12.9 years) [28], alpha-mannosidosis (72 months) [29], adult hypophosphatasia (over 3.8 years) [30], Farber’s disease (13.7 months) [31], and mucopolysaccharidosis type III (39 months) [32]. This delay in the diagnosis of rare metabolic diseases is more common among those that do not form part of neonatal screening or do not exhibit altered levels of biochemical parameters in routine biological samples [33]. In contrast, a time to diagnosis of less than a year is reported for cardiac amyloidosis (10 months) [34], mucopolysaccharidosis type I (9 months) [32] and childhood hypophosphatasia (8.4 weeks) [30].

In general, in addition to metabolic diseases which are diagnosed in neonatal screening, diseases of paediatric appearance (under age 15 years) are usually diagnosed before they present at adult age. For instance, at a paediatric level, congenital malformations predominate (39.1% of diseases of paediatric appearance), with the patients in question registering a 48.4% diagnostic delay in our study. Among adults, diseases that affect the nervous system are more frequent (35.8% of diseases of adult appearance), and patients in this group register a 64.2% delay. Regarding sex, differences in diagnostic delay have been found. Specifically, diagnostic delay is higher in women than in men in musculoskeletal, endocrine and eye and adnexa diseases. This gender gap is also observed in other studies with common diseases in which women are diagnoses later than men [35].

The difficulty of diagnosing certain RDs is due to the fact that many of them do not have specific tests and present with widely varying phenotypical manifestations [36]. In the case of RDs identified with diagnostic delay, it should be noted that such delay is especially harmful for patients affected by Usher Syndrome (our study indicates a diagnostic delay of over 75%), muscular dystrophies (Becker, Duchenne or facioescapulohumeral), Friedreich ataxia, lymphangioleiomyomatosis, hereditary angio-oedema, amyotrophic lateral sclerosis, or acquired epidermolysis bullosa. These RDs, among others, highlight the urgent need for development of solutions that would serve to reduce the delay in diagnosis [37].

In terms of time trend, the percentage of persons who experienced diagnostic delays decreased across the period between the 1960s and the present. This decrease is consistent with scientific and medical advances, increased knowledge of RDs, and improvements in diagnostic techniques. In this respect, the discovery of Next Generation Sequencing has radically changed diagnosis of certain genetic RDs, in that this is a fast, powerful and increasingly available alternative which has reduced waiting times [33]. The marked fall seen from 2018 onwards may be affected by the fact that all the study participants were persons already diagnosed with a RD, meaning that those with recent symptom onset have not yet had the opportunity to be diagnosed and included in this study. Given that mean time to diagnosis is 6 years, it would be more appropriate to pay attention to the trend registered until 2018, which shows a gradual decrease in the percentage of patients who experienced diagnostic delays in Spain. When it comes to Autonomous Regions, it should be stressed that while no evidence was found to show that diagnostic delays might vary by region, more in-depth study on this topic is nevertheless called for, since it is a matter of concern to patient associations.

It is clear that the diagnostic delay of RD is a public health problem. Therefore, it would be advisable to establish some potential measures like improving: the scientific knowledge of RDs; the accessibility to different clinicians o centres of references (sometimes by shortening the distance among them); the time spent in attending the different medical appointments; the availability of ad hoc diagnostic tests; the capacity of reffering to other centres easily or the increase of staff. In Spain there are 296 Reference Centres, Services and Units in the National Health System. They are distributed in 52 centres, includes 70 pathologies and many of them are part of the European Reference Networks. However, these centres do not cover all RD as many patients´ organizations claim.

Mention should be made of the difficulty of validating the variable, ‘time to diagnosis’. The limitations on being able to establish both disease onset and the fact that diagnosis was validly made are usual in these types of studies, if this information is not specifically recorded in the clinical report. If the first manifestations are clearly attributable to the disease, the date of symptom onset can be estimated, especially if it is connected to events in the family’s social life. The date of diagnosis can occur: (a) when it is linked to an analytical, histological or imaging test that allows for no doubt; or alternatively, (b) within the framework of a diagnostic process that is long per se. In such a case, diagnosis can be obtained with a high level of certainty in a given time, but confirmation may be altogether slower in coming, due to need to perform techniques that take time. Hence, whereas some patients might consider themselves to be already diagnosed on the basis of clinical suspicion, others would not venture to do so until receipt of the final confirmatory test, even though this may well corroborate the clinical diagnosis. These problems are further aggravated in the case of diagnoses made decades ago, though the general trend among patients insofar as it might serve to increase or decrease this time is not a systematic problem. In this study an effort was made to use the information stated in clinical reports, submitted by health professionals through medical societies, and/or recorded in the registry. Lastly, the lack of a population-based registry for RDs as a whole means that the distribution of all patients and all RDs in a specific geographical region cannot be properly ascertained. As a result, these aspects could limit extrapolation of the results obtained to RDs as whole.

Despite the limitations, the main strength of this study is that it is the first to quantify time to diagnosis of RDs in Spain, based on information sourced from a national registry open to any RD. Another point to be highlighted is that, in comparison with the only previous studies to furnish data on diagnostic delays in general for RDs in Spain [8, 38], our study not only relied on information covering a greater number of diseases and persons affected, confirmed diagnoses of RD, and a heterogeneous population in socio-demographic terms, but the data were also gathered over a long period of time.

## Conclusion

This is the first study to quantify the time taken to arrive at a diagnosis, as well as diagnostic delays for RDs as a whole, based on the Spanish Rare Diseases Patient Registry. The data reported corroborate the fact that more than half of all persons affected by RDs in Spain experience diagnostic delays, and that, despite the reduction achieved since the 1960s, the country is still far from reaching the IRDiRC goal of obtaining a diagnosis within less than a year. It is therefore essential to continue monitoring time to diagnosis, while at the same time conducting in-depth studies to ascertain the factors associated with delay and the implications that all this has for every sphere of the lives of patients and their families, in view of the social and health problems involved.

## Supplementary Information

**Additional file 1. Table S1**. Complete list of included RD and their ORPHAcodes (version 2021).

## Data Availability

Not applicable.

## Abbreviations

APC: Annual percent change
IRDiRC: International Rare Diseases Research Consortium
RD: Rare diseases
FEDER: Spanish Federation of Rare Diseases
ISCIII: Institute of Health Carlos III
IIER: Institute of Rare Diseases Research
ICD: International Classification of Diseases
IQR: Interquartile range
NS: Non-significant difference
OMIM: Online Mendelian Inheritance in Man
OR: Odds ratio
