# Supplementary material for: Diagnostic delay in rare diseases: data from the Spanish rare diseases patient registry
Source: Orphanet J Rare Dis. 2022 Nov 17;17:418. doi: 10.1186/s13023-022-02530-3 (PMC9670379; doi:10.1186/s13023-022-02530-3)
Supplement: Supplementary file 1 — Additional file 1. Table S1. Complete list of included RD and their ORPHAcodes (version 2021). [file 13023_2022_2530_MOESM1_ESM.docx]

Additional file 1: Table S1. Complete list of included RD and their ORPHAcodes (version 2021)

| ORPHAcode | Disease name |
| --- | --- |
| 199318 | 15q13.3 microdeletion syndrome |
| 96078 | 16p13.3 microduplication syndrome |
| 1713 | 17p11.2 microduplication syndrome |
| 261272 | 17q12 microduplication syndrome |
| 1606 | 1p36 deletion síndrome |
| 567 | 22q11.2 deletion syndrome |
| 251019 | 2q32q33 microdeletion síndrome |
| 65286 | 3q29 microdeletion syndrome |
| 96072 | 4p16.3 microduplication syndrome |
| 96121 | 7q11.23 microduplication syndrome |
| 251071 | 8p23.1 microdeletion síndrome |
| 284160 | 8q21.11 microdeletion syndrome |
| 531151 | 9q21.13 microdeletion syndrome |
| 15 | Achondroplasia |
| 49382 | Achromatopsia |
| 950 | Acrodysostosis |
| 963 | Acromegaly |
| 79276 | Acute intermittent porphyria |
| 85138 | Addison disease |
| 45 | Adenosine monophosphate deaminase deficiency |
| 404448 | ADNP syndrome |
| 247676 | Adult hypophosphatasia |
| 2688 | Adult idiopathic neutropenia |
| 206583 | Adult polyglucosan body disease |
| 99000 | Adult-onset foveomacular vitelliform dystrophy |
| 829 | Adult-onset Still disease |
| 50 | Aicardi syndrome |
| 51 | Aicardi-Goutières syndrome |
| 52 | Alagille syndrome |
| 261619 | Alagille syndrome due to a JAG1 point mutation |
| 56 | Alkaptonuria |
| 59 | Allan-Herndon-Dudley syndrome |
| 701 | Alopecia universalis |
| 726 | Alpers-Huttenlocher syndrome |
| 60 | Alpha-1-antitrypsin deficiency |
| 62 | Alpha-sarcoglycan-related limb-girdle muscular dystrophy R3 |
| 64 | Alström syndrome |
| 2131 | Alternating hemiplegia of childhood |
| 3386 | American trypanosomiasis |
| 803 | Amyotrophic lateral sclerosis |
| 72 | Angelman syndrome |
| 1071 | Ankyloblepharon-ectodermal defects-cleft lip/palate syndrome |
| 77298 | Anophthalmia/microphthalmia-esophageal atresia syndrome |
| 96346 | Anorectal malformation |
| 80 | Antiphospholipid syndrome |
| 81 | Antisynthetase syndrome |
| 87 | Apert syndrome |
| 2356 | Arachnoid cyst |
| 137817 | Arachnoiditis |
| 268882 | Arnold-Chiari malformation type I |
| 1136 | Arnold-Chiari malformation type II |
| 1037 | Arthrogryposis multiplex congenita |
| 100 | Ataxia-telangiectasia |
| 85451 | ATTRV122I amyloidosis |
| 2134 | Atypical hemolytic uremic syndrome |
| 352490 | Autism spectrum disorder due to AUTS2 deficiency |
| 2137 | Autoimmune hepatitis |
| 103919 | Autoimmune pancreatitis |
| 3453 | Autoimmune polyendocrinopathy type 1 |
| 3143 | Autoimmune polyendocrinopathy type 2 |
| 747 | Autoimmune pulmonary alveolar proteinosis |
| 99 | Autosomal dominant cerebellar ataxia |
| 208508 | Autosomal dominant cerebellar ataxia type II |
| 99944 | Autosomal dominant Charcot-Marie-Tooth disease type 2K |
| 1432 | Autosomal dominant chorioretinopathy-microcephaly syndrome |
| 98853 | Autosomal dominant Emery-Dreifuss muscular dystrophy |
| 312 | Autosomal dominant epidermolytic ichthyosis |
| 457193 | Autosomal dominant intellectual disability-craniofacial anomalies-cardiac defects syndrome |
| 178469 | Autosomal dominant non-syndromic intellectual disability |
| 100984 | Autosomal dominant spastic paraplegia type 3 |
| 100985 | Autosomal dominant spastic paraplegia type 4 |
| 79278 | Autosomal erythropoietic protoporphyria |
| 91024 | Autosomal recessive axonal hereditary motor and sensory neuropathy |
| 1172 | Autosomal recessive cerebellar ataxia |
| 284282 | Autosomal recessive cerebellar ataxia-epilepsy-intellectual disability syndrome due to WWOX deficiency |
| 1170 | Autosomal recessive cerebelloparenchymal disorder type 3 |
| 98855 | Autosomal recessive Emery-Dreifuss muscular dystrophy |
| 98 | Autosomal recessive spastic ataxia of Charlevoix-Saguenay |
| 2822 | Autosomal recessive spastic paraplegia type 11 |
| 100996 | Autosomal recessive spastic paraplegia type 15 |
| 488594 | Autosomal recessive spastic paraplegia type 76 |
| 782 | Axenfeld-Rieger syndrome |
| 110 | Bardet-Biedl syndrome |
| 98895 | Becker muscular dystrophy |
| 117 | Behçet disease |
| 251287 | Benign concentric annular macular dystrophy |
| 274 | Bernard-Soulier syndrome |
| 1243 | Best vitelliform macular dystrophy |
| 610 | Bethlem myopathy |
| 41751 | Bietti crystalline dystrophy |
| 208447 | Bilateral generalized polymicrogyria |
| 1980 | Bilateral striopallidodentate calcinosis |
| 364198 | Bipartite talus |
| 122 | Birt-Hogg-Dubé syndrome |
| 124 | Blackfan-Diamond anemia |
| 93930 | Bladder exstrophy |
| 90340 | Blau syndrome |
| 93964 | Blepharospasm-oromandibular dystonia syndrome |
| 16 | Blue cone monochromatism |
| 97297 | Bohring-Opitz syndrome |
| 127 | Borjeson-Forssman-Lehmann syndrome |
| 90354 | Brittle cornea syndrome |
| 1303 | Bronchiolitis obliterans with obstructive pulmonary disease |
| 130 | Brugada syndrome |
| 36258 | Buerger disease |
| 703 | Bullous pemphigoid |
| 135 | CACH syndrome |
| 267 | Calpain-3-related limb-girdle muscular dystrophy R1 |
| 1340 | Cardiofaciocutaneous syndrome |
| 157 | Carnitine palmitoyltransferase II deficiency |
| 53035 | Caroli disease |
| 175 | Cartilage-hair hypoplasia |
| 3286 | Catecholaminergic polymorphic ventricular tachycardia |
| 3027 | Caudal regression sequence |
| 75377 | Central areolar choroidal dystrophy |
| 597 | Central core disease |
| 178029 | Central diabetes insipidus |
| 504476 | Cerebellar ataxia with neuropathy and bilateral vestibular areflexia syndrome |
| 46724 | Cerebral arteriovenous malformation |
| 136 | Cerebral autosomal dominant arteriopathy-subcortical infarcts-leukoencephalopathy |
| 101081 | Charcot-Marie-Tooth disease type 1A |
| 99948 | Charcot-Marie-Tooth disease type 4A |
| 166 | Charcot-Marie-Tooth disease/Hereditary motor and sensory neuropathy |
| 138 | CHARGE syndrome |
| 599082 | CHD3-related developmental delay-speech delay-intellectual disability-abnormalities of vision-facial dysmorphism syndrome |
| 75234 | Cholesteryl ester storage disease |
| 180 | Choroideremia |
| 2932 | Chronic inflammatory demyelinating polyneuropathy |
| 91359 | Chronic pneumonitis of infancy |
| 600731 | Clark-Baraitser syndrome |
| 93605 | Classic Bartter syndrome |
| 90794 | Classic congenital adrenal hyperplasia due to 21-hydroxylase deficiency |
| 79239 | Classic galactosemia |
| 71277 | Classic glucose transporter type 1 deficiency syndrome |
| 2584 | Classic mycosis fungoides |
| 1452 | Cleidocranial dysplasia |
| 93929 | Cloacal exstrophy |
| 313838 | Coats plus syndrome |
| 192 | Coffin-Lowry syndrome |
| 1465 | Coffin-Siris syndrome |
| 1467 | Cogan syndrome |
| 193 | Cohen syndrome |
| 98942 | Coloboma of choroid and retina |
| 98944 | Coloboma of iris |
| 98203 | Combined dystonia |
| 444013 | Combined oxidative phosphorylation defect type 23 |
| 95494 | Combined pituitary hormone deficiencies, genetic forms |
| 1572 | Common variable immunodeficiency |
| 280821 | Communicating congenital bronchopulmonary-foregut malformation |
| 99995 | Complex regional pain syndrome type 1 |
| 99994 | Complex regional pain syndrome type 2 |
| 1872 | Cone rod dystrophy |
| 95499 | Congenital anomaly of the inferior vena cava |
| 79277 | Congenital erythropoietic porphyria |
| 327 | Congenital factor VII deficiency |
| 329 | Congenital factor XI deficiency |
| 2020 | Congenital fiber-type disproportion myopathy |
| 45358 | Congenital fibrosis of extraocular muscles |
| 98976 | Congenital glaucoma |
| 2185 | Congenital hydrocephalus |
| 442 | Congenital hypothyroidism |
| 562528 | Congenital limbs-face contractures-hypotonia-developmental delay syndrome |
| 97245 | Congenital myopathy |
| 79394 | Congenital non-bullous ichthyosiform erythroderma |
| 2414 | Congenital pulmonary lymphangiectasia |
| 103908 | Congenital sodium diarrhea |
| 141127 | Congenital tracheal stenosis |
| 98396 | Constitutional megaloblastic anemia due to vitamin B12 metabolism disorder |
| 725 | Continuous spikes and waves during sleep |
| 199 | Cornelia de Lange syndrome |
| 3071 | Costello syndrome |
| 201 | Cowden syndrome |
| 90290 | CREST syndrome |
| 1545 | Crisponi syndrome |
| 96253 | Cushing disease |
| 66646 | Cutaneous mastocytosis |
| 209 | Cutis laxa |
| 586 | Cystic fibrosis |
| 213 | Cystinosis |
| 64748 | Dejerine-Sottas syndrome |
| 1652 | Dent disease |
| 221 | Dermatomyositis |
| 98909 | Desminopathy |
| 873 | Desmoid tumor |
| 79456 | Diffuse cutaneous mastocytosis |
| 2123 | Diffuse neonatal hemangiomatosis |
| 90281 | Discoid lupus erythematosus |
| 308463 | Disorder of fructose metabolism |
| 79167 | Disorder of urea cycle metabolism and ammonia detoxification |
| 96149 | Distal monosomy 12q |
| 36367 | Distal monosomy 1q |
| 1620 | Distal monosomy 3p |
| 98911 | Distal myotilinopathy |
| 18 | Distal renal tubular acidosis |
| 1707 | Distal trisomy 15q |
| 33069 | Dravet síndrome |
| 233 | Duane retraction syndrome |
| 98896 | Duchenne muscular dystrophy |
| 97339 | Dural sinus malformation |
| 268 | Dysferlin-related limb-girdle muscular dystrophy R2 |
| 303 | Dystrophic epidermolysis bullosa |
| 1934 | Early infantile epileptic encephalopathy |
| 1177 | Early-onset cerebellar ataxia with retained tendon reflexes |
| 488635 | Early-onset epilepsy-intellectual disability-brain anomalies syndrome |
| 256 | Early-onset generalized limb-onset dystonia |
| 91492 | Early-onset non-syndromic cataract |
| 158668 | Ectodermal dysplasia-skin fragility syndrome |
| 498477 | Ectrodactyly with and without other manifestations |
| 98249 | Ehlers-Danlos syndrome |
| 97214 | Eisenmenger syndrome |
| 289 | Ellis Van Creveld syndrome |
| 96170 | Emanuel syndrome |
| 73247 | Eosinophilic esophagitis |
| 3165 | Eosinophilic fasciitis |
| 2070 | Eosinophilic gastroenteritis |
| 183 | Eosinophilic granulomatosis with polyangiitis |
| 304 | Epidermolysis bullosa simplex |
| 157791 | Epithelioid hemangioendothelioma |
| 1199 | Esophageal atresia |
| 98981 | Essential iris atrophy |
| 3318 | Essential thrombocythemia |
| 51188 | Ethylmalonic encephalopathy |
| 3023 | External auditory canal atresia-vertical talus-hypertelorism syndrome |
| 100054 | F12-related hereditary angioedema with normal C1Inh |
| 324 | Fabry disease |
| 85162 | Facial onset sensory and motor neuronopathy |
| 269 | Facioscapulohumeral dystrophy |
| 2841 | Familial benign chronic pemphigus |
| 75376 | Familial drusen |
| 891 | Familial exudative vitreoretinopathy |
| 342 | Familial Mediterranean fever |
| 97 | Familial paroxysmal ataxia |
| 31043 | Familial primary hypomagnesemia with hypercalciuria and nephrocalcinosis without severe ocular involvement |
| 84 | Fanconi anemia |
| 466 | Fatal familial insomnia |
| 163703 | Febrile infection-related epilepsy syndrome |
| 101039 | Female restricted epilepsy with intellectual disability |
| 337 | Fibrodysplasia ossificans progressiva |
| 1866 | Focal, segmental or multifocal dystonia |
| 561854 | FOXG1 syndrome |
| 908 | Fragile X syndrome |
| 95 | Friedreich ataxia |
| 79665 | Gardner syndrome |
| 44890 | Gastrointestinal stromal tumor |
| 77259 | Gaucher disease type 1 |
| 358 | Gitelman syndrome |
| 849 | Glanzmann thrombasthenia |
| 488613 | Global developmental delay-neuro-ophthalmological abnormalities-seizures-intellectual disability syndrome |
| 25 | Glutaryl-CoA dehydrogenase deficiency |
| 365 | Glycogen storage disease due to acid maltase deficiency |
| 79259 | Glycogen storage disease due to glucose-6-phosphatase deficiency type Ib |
| 366 | Glycogen storage disease due to glycogen debranching enzyme deficiency |
| 368 | Glycogen storage disease due to muscle glycogen phosphorylase deficiency |
| 370 | Glycogen storage disease due to phosphorylase kinase deficiency |
| 354 | GM1 gangliosidosis |
| 602 | GNE myopathy |
| 374 | Goldenhar syndrome |
| 1532 | Gómez-López-Hernández syndrome |
| 377 | Gorlin syndrome |
| 900 | Granulomatosis with polyangiitis |
| 2103 | Guillain-Barré syndrome |
| 414 | Gyrate atrophy of choroid and retina |
| 2108 | Hallermann-Streiff syndrome |
| 221083 | Hemifacial spasm |
| 766 | Hemolytic anemia due to red cell pyruvate kinase deficiency |
| 98878 | Hemophilia A |
| 91378 | Hereditary angioedema |
| 100050 | Hereditary angioedema type 1 |
| 100051 | Hereditary angioedema type 2 |
| 79273 | Hereditary coproporphyria |
| 469 | Hereditary fructose intolerance |
| 774 | Hereditary hemorrhagic telangiectasia |
| 523 | Hereditary leiomyomatosis and renal cell cancer |
| 64751 | Hereditary motor and sensory neuropathy type 5 |
| 640 | Hereditary neuropathy with liability to pressure palsies |
| 685 | Hereditary spastic paraplegia |
| 189 | Hidrotic ectodermal dysplasia |
| 84085 | Hinman syndrome |
| 388 | Hirschsprung disease |
| 454718 | Holmes-Adie syndrome |
| 2162 | Holoprosencephaly |
| 392 | Holt-Oram syndrome |
| 2744 | Horizontal gaze palsy with progressive scoliosis |
| 399 | Huntington disease |
| 343 | Hyperimmunoglobulinemia D with periodic fever |
| 411 | Hyperlipoproteinemia type 1 |
| 285 | Hypermobile Ehlers-Danlos syndrome |
| 238468 | Hypohidrotic ectodermal dysplasia |
| 681 | Hypokalemic periodic paralysis |
| 79233 | Hypoxanthine guanine phosphoribosyltransferase partial deficiency |
| 930 | Idiopathic achalasia |
| 238624 | Idiopathic intracranial hypertension |
| 570431 | Idiopathic multicentric Castleman disease |
| 494428 | Idiopathic pleuroparenchymal fibroelastosis |
| 275766 | Idiopathic pulmonary arterial hypertension |
| 2032 | Idiopathic pulmonary fibrosis |
| 99931 | Idiopathic pulmonary hemosiderosis |
| 49041 | IgG4-related retroperitoneal fibrosis |
| 3002 | Immune thrombocytopenia |
| 52430 | Inclusion body myopathy with Paget disease of bone and frontotemporal dementia |
| 611 | Inclusion body myositis |
| 98848 | Indolent systemic mastocytosis |
| 3451 | Infantile spasms syndrome |
| 2298 | Insulin-resistance syndrome type B |
| 464311 | Intellectual disability syndrome due to a DYRK1A point mutation |
| 3042 | Intellectual disability-cataracts-calcified pinnae-myopathy syndrome |
| 329224 | Intellectual disability-craniofacial dysmorphism-cryptorchidism syndrome |
| 436151 | Intellectual disability-expressive aphasia-facial dysmorphism syndrome |
| 404440 | Intellectual disability-facial dysmorphism syndrome due to SETD5 haploinsufficiency |
| 457279 | Intellectual disability-macrocephaly-hypotonia-behavioral abnormalities syndrome |
| 37202 | Interstitial cistitis |
| 3306 | Inverted duplicated chromosome 15 syndrome |
| 597623 | IRF2BPL-related regressive neurodevelopmental disorder-dystonia-seizures syndrome |
| 84142 | Isaac síndrome |
| 250923 | Isolated aniridia |
| 30391 | Isolated biliary atresia |
| 2609 | Isolated complex I deficiency |
| 1460 | Isolated complex III deficiency |
| 238666 | Isolated congenital hypogonadotropic hypogonadism |
| 200 | Isolated corpus callosum agenesis |
| 254905 | Isolated cytochrome C oxidase deficiency |
| 2345 | Isolated Klippel-Feil syndrome |
| 35093 | Isolated scaphocephaly |
| 3208 | Isolated succinate-CoQ reductase deficiency |
| 2308 | Jacobsen syndrome |
| 475 | Joubert syndrome |
| 93672 | Juvenile dermatomyositis |
| 79264 | Juvenile neuronal ceroid lipofuscinosis |
| 329894 | Juvenile overlap myositis |
| 158000 | Juvenile xanthogranuloma |
| 2322 | Kabuki syndrome |
| 478 | Kallmann syndrome |
| 2332 | KBG syndrome |
| 439218 | KCNQ2-related epileptic encephalopathy |
| 480 | Kearns-Sayre syndrome |
| 481 | Kennedy disease |
| 97332 | Kienbock disease |
| 482 | Kimura disease |
| 33543 | Kleine-Levin syndrome |
| 90308 | Klippel-Trénaunay syndrome |
| 485 | Kniest dysplasia |
| 96169 | Koolen-De Vries syndrome |
| 487 | Krabbe disease |
| 530983 | Lamb-Shaffer syndrome |
| 313 | Lamellar ichthyosis |
| 258 | Laminin subunit alpha 2-related congenital muscular dystrophy |
| 98818 | Landau-Kleffner syndrome |
| 389 | Langerhans cell histiocytosis |
| 626 | Large congenital melanocytic nevus |
| 503 | Larsen syndrome |
| 168491 | Late infantile neuronal ceroid lipofuscinosis |
| 65 | Leber congenital amaurosis |
| 104 | Leber hereditary optic neuropathy |
| 2380 | Legg-Calvé-Perthes disease |
| 506 | Leigh syndrome |
| 64720 | Leiomyosarcoma |
| 510 | Lesch-Nyhan syndrome |
| 68356 | Leukodystrophy |
| 263 | Limb-girdle muscular dystrophy |
| 220402 | Limited cutaneous systemic sclerosis |
| 158673 | Localized dystrophic epidermolysis bullosa, acral form |
| 90289 | Localized scleroderma |
| 60030 | Loeys-Dietz syndrome |
| 5 | Long chain 3-hydroxyacyl-CoA dehydrogenase deficiency |
| 168 | Loose anagen syndrome |
| 91546 | Lyme disease |
| 538 | Lymphangioleiomyomatosis |
| 98842 | Lymphomatoid papulosis |
| 144 | Lynch syndrome |
| 79489 | Macrocystic lymphatic malformation |
| 558 | Marfan syndrome |
| 59306 | McLeod neuroacanthocytosis syndrome |
| 42 | Medium chain acyl-CoA dehydrogenase deficiency |
| 2478 | Megalencephalic leukoencephalopathy with subcortical cysts |
| 2477 | Megalencephaly |
| 60040 | Megalencephaly-capillary malformation-polymicrogyria syndrome |
| 90186 | Meige disease |
| 550 | MELAS |
| 2483 | Melkersson-Rosenthal syndrome |
| 2485 | Melorheostosis |
| 93968 | Meningocele |
| 592574 | Menke-Hennekam syndrome |
| 565 | Menkes disease |
| 551 | MERRF |
| 99701 | Mesial temporal lobe epilepsy with hippocampal sclerosis |
| 512 | Metachromatic leukodystrophy |
| 33067 | Metaphyseal chondrodysplasia, Jansen type |
| 79282 | Methylmalonic acidemia with homocystinuria, type cblC |
| 231736 | Microcornea-posterior megalolenticonus-persistent fetal vasculature-coloboma syndrome |
| 531 | Miller-Dieker syndrome |
| 79452 | Milroy disease |
| 35698 | Mitochondrial DNA depletion syndrome |
| 663 | Mitochondrial DNA-related progressive external ophthalmoplegia |
| 206966 | Mitochondrial myopathy |
| 2597 | Mitochondrial myopathy-lactic acidosis-deafness syndrome |
| 809 | Mixed connective tissue disease |
| 45448 | Miyoshi myopathy |
| 52368 | Mohr-Tranebjaerg syndrome |
| 529468 | Monoclonal mast cell activation syndrome |
| 65684 | Monomelic amyotrophy |
| 1598 | Monosomy 18p |
| 48652 | Monosomy 22q13.3 |
| 281 | Monosomy 5p |
| 261112 | Monosomy 9p |
| 93277 | Monostotic fibrous dysplasia |
| 99228 | Mosaic monosomy X |
| 98503 | Motor neuron disease |
| 2573 | Moyamoya disease |
| 575 | Muckle-Wells syndrome |
| 580 | Mucopolysaccharidosis type 2 |
| 581 | Mucopolysaccharidosis type 3 |
| 582 | Mucopolysaccharidosis type 4 |
| 2774 | Multicentric carpo-tarsal osteolysis with or without nephropathy |
| 598 | Multiminicore myopathy |
| 26791 | Multiple acyl-CoA dehydrogenase deficiency |
| 247698 | Multiple endocrine neoplasia type 2ª |
| 247709 | Multiple endocrine neoplasia type 2B |
| 251 | Multiple epiphyseal dysplasia |
| 29073 | Multiple myeloma |
| 321 | Multiple osteochondromas |
| 102 | Multiple system atrophy |
| 227510 | Multiple system atrophy, cerebellar type |
| 98473 | Muscular dystrophy |
| 589 | Myasthenia gravis |
| 93969 | Myelomeningocele |
| 2588 | Myhre syndrome |
| 36899 | Myoclonus-dystonia syndrome |
| 178493 | Myopic macular degeneration |
| 2073 | Narcolepsy type 1 |
| 83465 | Narcolepsy type 2 |
| 644 | NARP syndrome |
| 607 | Nemaline myopathy |
| 223 | Nephrogenic diabetes insipidus |
| 2901 | Neuralgic amyotrophy |
| 263440 | Neuroacanthocytosis |
| 636 | Neurofibromatosis type 1 |
| 637 | Neurofibromatosis type 2 |
| 71211 | Neuromyelitis optica spectrum disorder |
| 3051 | Nicolaides-Baraitser syndrome |
| 77293 | Niemann-Pick disease type B |
| 646 | Niemann-Pick disease type C |
| 90695 | Non-acquired panhypopituitarism |
| 217629 | Non-familial dilated cardiomyopathy |
| 442835 | Non-specific early-onset epileptic encephalopathy |
| 91364 | Non-specific interstitial pneumonia |
| 648 | Noonan syndrome |
| 500 | Noonan syndrome with multiple lentigines |
| 2701 | Noonan syndrome-like disorder with loose anagen hair |
| 649 | Norrie disease |
| 600663 | NRXN1-related severe neurodevelopmental disorder-motor stereotypies-chronic constipation-sleep-wake cycle disturbance |
| 284804 | Ocular albinism |
| 534 | Oculocerebrorenal syndrome of Lowe |
| 55 | Oculocutaneous albinism |
| 270 | Oculopharyngeal muscular dystrophy |
| 296 | Ollier disease |
| 661 | Ondine syndrome |
| 1183 | Opsoclonus-myoclonus syndrome |
| 401777 | Optic atrophy-intellectual disability syndrome |
| 93958 | Oromandibular dystonia |
| 2764 | Osteochondritis dissecans |
| 666 | Osteogenesis imperfecta |
| 216820 | Osteogenesis imperfecta type 4 |
| 251312 | Overlapping connective tissue disease |
| 363478 | Paratesticular adenocarcinoma |
| 157835 | Paroxysmal hemicrania |
| 447 | Paroxysmal nocturnal hemoglobinuria |
| 262146 | Partial deletion of the long arm of chromosome 18 |
| 262010 | Partial deletion of the long arm of chromosome 2 |
| 262083 | Partial monosomy of the long arm of chromosome 10 |
| 262658 | Partial trisomy/tetrasomy of the short arm of chromosome 12 |
| 699 | Pearson syndrome |
| 702 | Pelizaeus-Merzbacher disease |
| 704 | Pemphigus vulgaris |
| 65250 | Perineural cyst |
| 79189 | Peroxisome biogenesis disorder |
| 708 | Peters anomaly |
| 2869 | Peutz-Jeghers syndrome |
| 716 | Phenylketonuria |
| 66627 | Pigmented villonodular synovitis |
| 2896 | Pitt-Hopkins syndrome |
| 91354 | Pituitary deficiency due to empty sella turcica syndrome |
| 251607 | Pleomorphic xanthoastrocytoma |
| 79318 | PMM2-CDG |
| 2905 | POEMS syndrome |
| 2911 | Poland syndrome |
| 767 | Polyarteritis nodosa |
| 729 | Polycythemia vera |
| 732 | Polymyositis |
| 79473 | Porphyria variegata |
| 88628 | Posterior column ataxia-retinitis pigmentosa syndrome |
| 279947 | Postorgasmic illness syndrome |
| 2942 | Postpoliomyelitis syndrome |
| 443236 | Postural orthostatic tachycardia syndrome due to NET deficiency |
| 52022 | Potocki-Shaffer syndrome |
| 739 | Prader-Willi syndrome |
| 186 | Primary biliary cholangitis |
| 244 | Primary ciliary dyskinesia |
| 98861 | Primary ciliary dyskinesia, Kartagener type |
| 90026 | Primary erythromelalgia |
| 314950 | Primary hypereosinophilic syndrome |
| 100049 | Primary interstitial lung disease specific to childhood due to pulmonary surfactant protein anomalies |
| 140436 | Primary intraosseous venous malformation |
| 35689 | Primary lateral sclerosis |
| 77240 | Primary lymphedema |
| 95432 | Primary progressive aphasia |
| 1871 | Progressive cone dystrophy |
| 1947 | Progressive epilepsy-intellectual disability syndrome, Finnish type |
| 1214 | Progressive hemifacial atrophy |
| 742 | Prolidase deficiency |
| 261197 | Proximal 16p11.2 microdeletion síndrome |
| 83330 | Proximal spinal muscular atrophy type 1 |
| 83418 | Proximal spinal muscular atrophy type 2 |
| 83419 | Proximal spinal muscular atrophy type 3 |
| 83420 | Proximal spinal muscular atrophy type 4 |
| 1762 | Proximal Xq28 duplication syndrome |
| 750 | Pseudoachondroplasia |
| 97593 | Pseudohypoparathyroidism |
| 79443 | Pseudohypoparathyroidism type 1A |
| 758 | Pseudoxanthoma elasticum |
| 60039 | Pudendal neuralgia |
| 60025 | Pulmonary alveolar microlithiasis |
| 275791 | Pulmonary arterial hypertension associated with another disease |
| 1207 | Pulmonary atresia with ventricular septal defect |
| 217557 | Pulmonary interstitial glycogenosis |
| 307967 | Punctate palmoplantar keratoderma |
| 441 | Pure autonomic failure |
| 763 | Pycnodysostosis |
| 71517 | Rapid-onset dystonia-parkinsonism |
| 391799 | Rare genetic dystonia |
| 183518 | Rare hereditary ataxia |
| 181405 | Rare hypoparathyroidism |
| 104012 | Rare inflammatory bowel disease |
| 71198 | Rare pulmonary hypertension |
| 29207 | Reactive arthritis |
| 461 | Recessive X-linked ichthyosis |
| 728 | Relapsing polychondritis |
| 71273 | Renal nutcracker syndrome |
| 335 | Reticular dysgenesis |
| 791 | Retinitis pigmentosa |
| 790 | Retinoblastoma |
| 778 | Rett syndrome |
| 85408 | Rheumatoid factor-negative polyarticular juvenile idiopathic arthritis |
| 177 | Rhizomelic chondrodysplasia punctata |
| 97229 | Riboflavin transporter deficiency |
| 96176 | Ring chromosome 13 syndrome |
| 1442 | Ring chromosome 18 syndrome |
| 1445 | Ring chromosome 21 syndrome |
| 2909 | Rothmund-Thomson syndrome |
| 3111 | Rotor syndrome |
| 783 | Rubinstein-Taybi syndrome |
| 793 | SAPHO syndrome |
| 797 | Sarcoidosis |
| 799 | Schizencephaly |
| 37748 | Schnitzler syndrome |
| 800 | Schwartz-Jampel syndrome |
| 331235 | Selective IgM deficiency |
| 100069 | Semantic dementia |
| 420402 | Semicircular canal dehiscence syndrome |
| 70595 | Sensory ataxic neuropathy-dysarthria-ophthalmoparesis syndrome |
| 35686 | Serpiginous choroiditis |
| 314911 | Severe Canavan disease |
| 280763 | Severe intellectual disability and progressive spastic paraplegia |
| 363686 | Severe intellectual disability-poor language-strabismus-grimacing face-long fingers syndrome |
| 404473 | Severe intellectual disability-progressive spastic diplegia syndrome |
| 811 | Shwachman-Diamond syndrome |
| 232 | Sickle cell anemia |
| 813 | Silver-Russell syndrome |
| 816 | Sjögren-Larsson syndrome |
| 819 | Smith-Magenis syndrome |
| 821 | Sotos syndrome |
| 99013 | Spastic paraplegia type 7 |
| 98757 | Spinocerebellar ataxia type 3 |
| 423296 | Spinocerebellar ataxia type 38 |
| 98766 | Spinocerebellar ataxia type 5 |
| 98758 | Spinocerebellar ataxia type 6 |
| 94147 | Spinocerebellar ataxia type 7 |
| 64753 | Spinocerebellar ataxia with axonal neuropathy type 2 |
| 94068 | Spondyloepiphyseal dysplasia congenita |
| 247234 | Sporadic adult-onset ataxia of unknown etiology |
| 3181 | Sprengel deformity |
| 827 | Stargardt disease |
| 273 | Steinert myotonic dystrophy |
| 83601 | Steroid-responsive encephalopathy associated with autoimmune thyroiditis |
| 36426 | Stevens-Johnson syndrome |
| 828 | Stickler syndrome |
| 3198 | Stiff person spectrum disorder |
| 98626 | Stromal corneal dystrophy |
| 98676 | Stromal corneal dystrophy |
| 3205 | Sturge-Weber syndrome |
| 22 | Succinic semialdehyde dehydrogenase deficiency |
| 306731 | Sydenham chorea |
| 79098 | Sympathetic ophthalmia |
| 199639 | Syndrome with corpus callosum agenesis/dysgenesis as a major feature |
| 544254 | SYNGAP1-related developmental and epileptic encephalopathy |
| 3280 | Syringomyelia |
| 188 | Systemic capillary leak syndrome |
| 536 | Systemic lupus erythematosus |
| 2467 | Systemic mastocytosis |
| 90291 | Systemic sclerosis |
| 85414 | Systemic-onset juvenile idiopathic arthritis |
| 3287 | Takayasu arteritis |
| 845 | Tay-Sachs disease |
| 884 | Tetrasomy 12p |
| 3310 | Tetrasomy 9p |
| 3312 | Thalidomide embryopathy |
| 614 | Thomsen and Becker disease |
| 97330 | Thoracic outlet syndrome |
| 54057 | Thrombotic thrombocytopenic purpura |
| 100100 | Thymic tumor |
| 42665 | Tietz síndrome |
| 64686 | Tolosa-Hunt syndrome |
| 98994 | Total early-onset cataract |
| 227972 | Toxic oil syndrome |
| 861 | Treacher-Collins syndrome |
| 77258 | Trichorhinophalangeal syndrome type 1 and 3 |
| 502 | Trichorhinophalangeal syndrome type 2 |
| 1209 | Tricuspid atresia |
| 221091 | Trigeminal neuralgia |
| 3380 | Trisomy 18 |
| 3375 | Trisomy X |
| 289326 | Tropical spastic paraparesis |
| 805 | Tuberous sclerosis complex |
| 32960 | Tumor necrosis factor receptor 1 associated periodic syndrome |
| 881 | Turner syndrome |
| 324936 | Unclassified autoinflammatory síndrome |
| 103920 | Undetermined colitis |
| 254837 | Unspecified mitochondrial disorder |
| 886 | Usher syndrome |
| 231169 | Usher syndrome type 1 |
| 231178 | Usher syndrome type 2 |
| 39044 | Uveal melanoma |
| 887 | VACTERL/VATER association |
| 286 | Vascular Ehlers-Danlos syndrome |
| 26793 | Very long chain acyl-CoA dehydrogenase deficiency |
| 252175 | Vestibular schwannoma |
| 892 | Von Hippel-Lindau disease |
| 166078 | Von Willebrand disease type 1 |
| 893 | WAGR syndrome |
| 899 | Walker-Warburg syndrome |
| 3452 | Whipple disease |
| 3207 | White matter hypoplasia-corpus callosum agenesis-intellectual disability syndrome |
| 904 | Williams syndrome |
| 905 | Wilson disease |
| 280 | Wolf-Hirschhorn syndrome |
| 3463 | Wolfram syndrome |
| 43 | X-linked adrenoleukodystrophy |
| 64747 | X-linked Charcot-Marie-Tooth disease |
| 52503 | X-linked creatine transporter deficiency |
| 777 | X-linked non-syndromic intellectual disability |
| 792 | X-linked retinoschisis |
| 86788 | X-linked severe congenital neutropenia |
| 910 | Xeroderma pigmentosum |
| 2828 | Young-onset Parkinson disease |

Note: There are other 54 RD that are not linked to a specific ORPHAcode.
